# A Large Ascending Aortic Aneurysm Secondary to Idiopathic Necrotizing Aortitis—A Rare but Important Cause of Thoracic Aortic Disease

**DOI:** 10.1055/s-0039-1693986

**Published:** 2019-11-22

**Authors:** Benjamin Smeeton, Muslim Mustaev, Michael Sabetai

**Affiliations:** 1 Department of Plastic & Reconstructive Surgery, St. George’s NHS Foundation Trust, London, United Kingdom; 2 Department of Cardiac Surgery, Guys' and St Thomas' NHS Foundation Trust, London, United Kingdom

**Keywords:** idiopathic necrotizing aortitis, aortic aneurysms, ascending aorta hemiarch replacement surgery, aortic valve replacement surgery

## Abstract

Idiopathic necrotizing aortitis is characterized by lymphoplasmacytic or giant cell-associated inflammation of the aorta, with no specific identifiable cause. We present the case of a 79-year-old man who sought medical attention from his primary care physician because of worsening shortness of breath. The patient underwent an elective ascending aorta, hemiarch, and aortic valve replacement. Histological examination of the aortic specimen demonstrated an unusually thin aorta with features consistent with necrotizing aortitis with giant cell infiltration.

## Introduction

Noninflammatory aortic diseases including cystic medial degeneration, atherosclerosis, and inherited connective tissue account for the majority of aortic aneurysms, whether thoracic or abdominal. Aortitis refers to a spectrum of disorders involving pathological inflammation of the aortic wall. 1 2 Although rare, aortitis represents an increasingly recognized cause of aneurysmal disease. It is associated with significant morbidity and mortality not only through aneurysm development but also through aortic wall rupture, dissection, and luminal narrowing. 3 Aortitis may be either infective or noninfective in nature. Noninfective causes are typically associated with large-vessel vasculitides, such as giant cell arteritis (GCA) and Takayasu arteritis (TA). Rarely, as in this case presentation, aortitis manifests as an isolated finding, either histologically in patients postaortic surgery or identified radiologically, most often by magnetic resonance imagining or computed tomography (CT) scan. 4

## Case Presentation

A 79-year-old man sought medical attention from his primary care physician because of worsening shortness of breath on exertion for the last 2 years, with a recent, sudden deterioration in symptoms. More specifically, the patient reported a decrease in exertional tolerance from 1 mile to 100 yards and nocturnal dyspnea with two pillow orthopnea. His medical history included hypertension, hypercholesterolemia, osteoarthritis, and obstructive sleep apnea. He was an ex-smoker with a 20 pack-year history. He possessed no significant family history of cardiac or rheumatological disease. Aside from hypertension, physical examination was unremarkable.

**Fig. 1:**
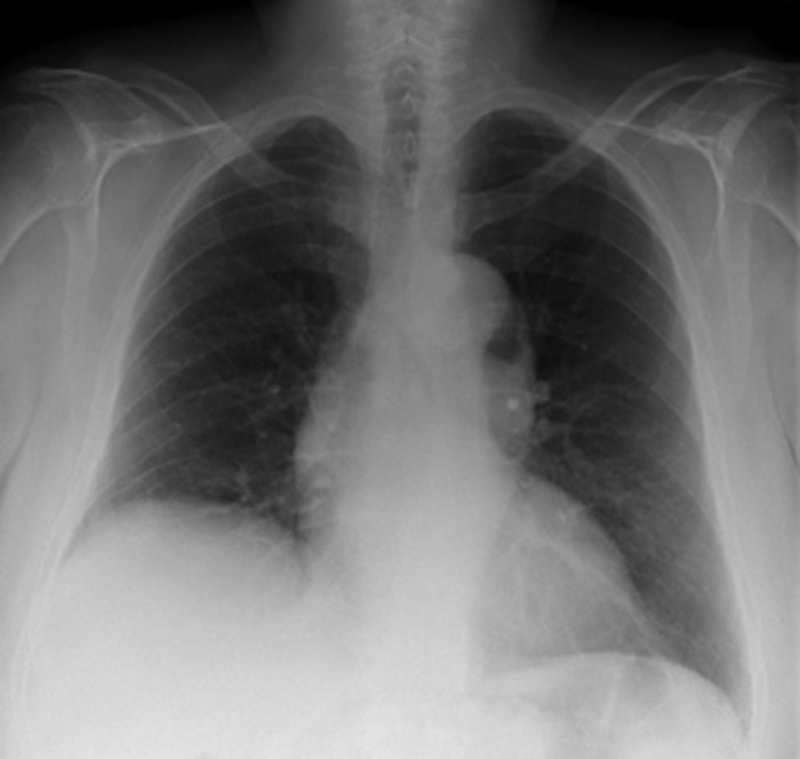
Anteroposterior chest radiograph demonstrating a widened mediastinum.

**Fig. 2:**
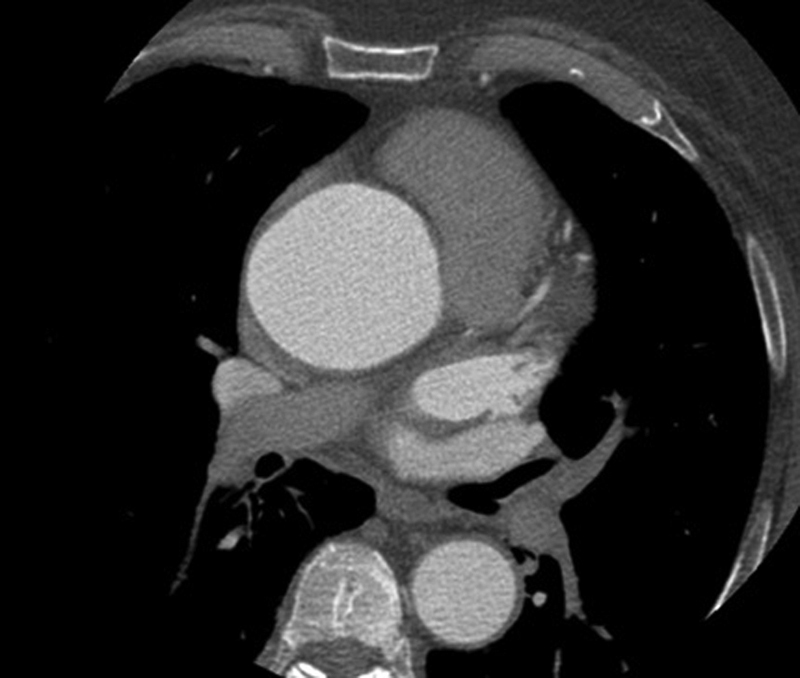
Axial section of aortic root demonstrating enlargement at the level of the sinotubular junction to 6.2 cm.

Initial laboratory tests were within normal ranges, including cardiac enzymes and inflammatory markers. An electrocardiogram was normal, although chest radiography demonstrated a widened mediastinum with a grossly dilated thoracic aorta ( Fig. 1 ). A contrast CT angiography scan of the thorax confirmed dilation of the ascending aorta, with a grossly dilated root with a maximum diameter of 62 mm ( Fig. 2 ). A transthoracic echocardiogram demonstrated moderate aortic regurgitation with preserved left ventricular function. Severe aortic root dilation with thinning of the aortic wall was also noted.

**Fig. 3:**
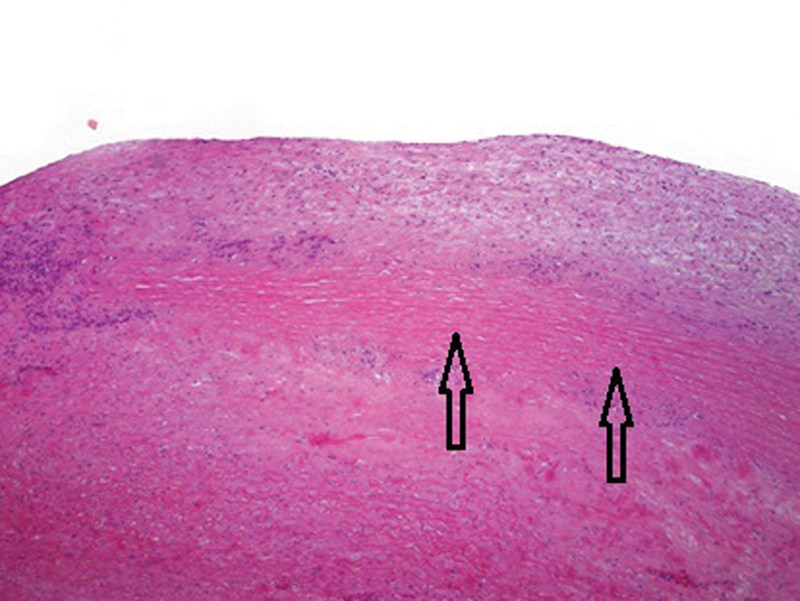
Histological sections from the resected aortic specimen stained with hematoxylin and eosin. 40X magnification demonstrates intima superiorly, underneath lies a band of necrotic media (elastic fibers are visible but no nuclei), with a layer of viable media beneath (shown by arrows).

**Fig. 4:**
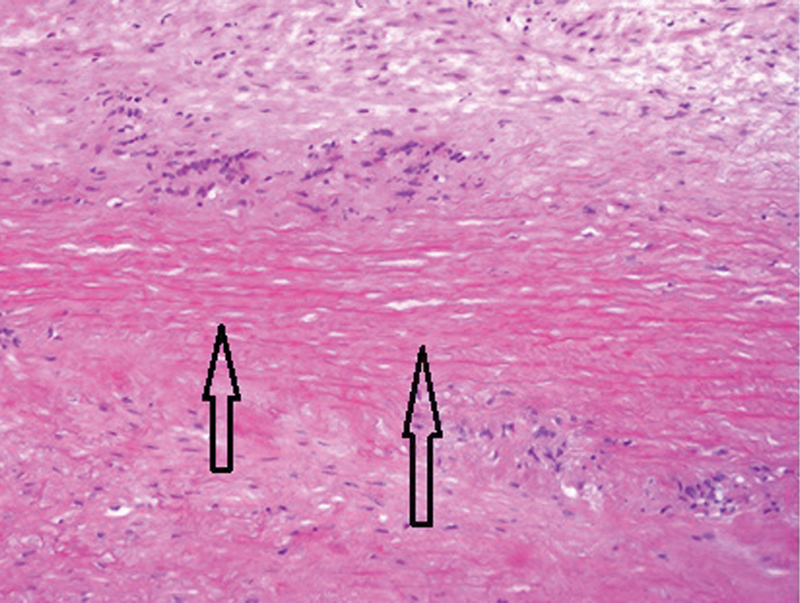
Histological sections from the resected aortic specimen stained with hematoxylin and eosin. 100X magnification demonstrates the necrotic band of media at higher power (shown by arrows). Histiocytes and occasional giant cells are visible at the periphery.

The patient underwent an elective aortic valve replacement with a 25-mm tissue valve and ascending aorta and hemiarch replacement with a 30-mm Dacron graft under hypothermic circulatory arrest. Histological examination of the aorta demonstrated patchy medial fibrosis, atrophy, and necrosis with occasional areas showing rimming of necrotic segments of media (laminar necrosis) by giant cells ( Figs. 3 and 4 ). There were neither neutrophilic infiltrates nor caseous-type necrosis. Examination was consistent with necrotizing aortitis with giant cells.

**Fig. 5:**
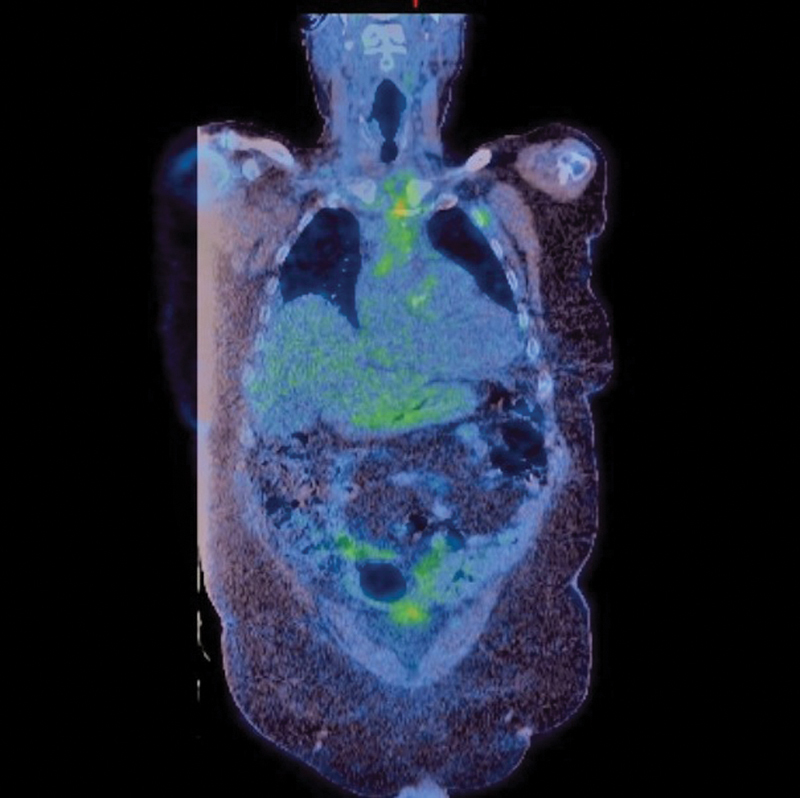
A postoperative half body fluorodeoxyglucose (FDG)-positron emission tomography scan demonstrates aortic valve and ascending aorta replacement with expected postsurgical change, with FDG avid mediastinal fat stranding and no convincing elevated FDG uptake in the wall of the native thoracic or abdominal aorta.

The differential for an isolated ascending aortitis includes inflammatory or infective processes. 2 4 Rheumatological serologic tests, including p- and c-anti-neutrophil cytoplasmic antibodies, Lyme antibody screen, total protein electrophoresis, and immunoglobulin G subclass analyses, were negative. A half body fluorodeoxyglucose-positron emission tomography scan was performed postoperatively, which suggested no active large vessel vasculitis ( Fig. 5 ). Infective causes were also excluded. Initial serology demonstrated a normal white cell count and inflammatory markers, and a subsequent blood culture was negative. Furthermore, histological analysis of the aortic specimen was not consistent with an infective process.

The patient had an unremarkable postoperative course and was discharged from the ward to be seen as an outpatient in 6 weeks, with a view to performing repeat cross-sectional imaging.

## Discussion

Although rare, aortitis is an increasingly recognized cause of thoracic aneurysmal disease. 3 If suspected, a thorough clinical assessment, including a detailed history and examination, with appropriate investigations is necessitated to exclude the potential of confounding infective or rheumatological processes. 2 4

Infective aortitis is a potentially life-threatening condition typically associated with bacterial or fungal infections. *Staphylococcus* and *Salmonella* tend to cause a pyogenic aortitis with acute sepsis and positive blood cultures. In contrast, syphilitic and mycobacterial aortitis manifest more insidiously. 2 5

Noninfective causes of aortitis include large-vessel vasculitides, such as GCA, TA, and Behçet's disease. Rarely, other systemic rheumatological disorders including systemic lupus erythematous, rheumatoid arthritis, and the human leukocyte antigen B27 (HLA-B27) spondyloarthropathies may also result in aortitis. 2 6 About 50 to 70% of patients with TA and up to 50% of those with GCA possess changes consistent with aortitis, many of whom progress to develop aneurysmal disease. 7 Other than biopsy, no specific tests exist in the diagnosis of GCA or TA, rather diagnosis is based on clinical criteria and imaging, as per the American College of Rheumatology. 5 8

In this case, the patient did not present symptoms or diagnostic criteria suggestive of an active vasculitis, or any other systemic inflammatory condition. Indeed, aortitis may be identified either histologically or radiologically in the absence of systemic disease. The so-called isolated aortitis rarely manifests with systemic symptoms; thus, it is highly likely that a proportion of these individuals never comes to medical attention. 2 4 In patients undergoing repair of aortic aneurysms, frequency of isolated aortitis ranges substantially from 1.7 to 8.8%. Higher incidence is observed in older patients and in those whom disease is restricted to the thoracic aorta. 3 6

Worryingly, it is becoming increasingly apparent that some patients initially diagnosed with isolated aortitis may actually possess a smoldering systemic vasculitis which may subsequently manifest at a later date. Potentially any systemic vasculitis that involves the aorta could manifest as an isolated aortitis. Although patients with large vessel vasculitides are now routinely screened for aortic involvement, an isolated aortitis may still represent the sole manifestation of a large vessel vasculitis. 2

A new diagnosis of noninfective aortitis warrants a full and thorough clinical evaluation for both additional arterial disease and symptoms suggestive of an underlying systemic inflammatory condition. A comprehensive review of the patient's medical records, an extensive patient history and examination, with particular focus on the vascular system, and appropriate laboratory testing should be performed. Furthermore, to determine if additional arterial lesions consistent with vasculitis are present, angiographic studies (typically magnetic resonance imaging or CT) of the aorta and its branches should be organized. Despite this, it is important to remember that since this single radiological snapshot may be unreliable in assessing overall disease activity in a large-vessel vasculitis, serial imaging should be conducted in all patients with radiologically suspected or histologically confirmed cases of aortitis. 7 8

Management remains controversial. For vasculitides such as TA and GCA, first-line treatment is with systemic corticosteroids, with chemotherapy agents such as cyclophosphamide reserved for severe cases. 6 However, in those postoperative patients in whom a diagnosis of an “isolated” aortitis has been established, the question remains: in whom is treatment required? With regard to immunosuppression, there are numerous conflicting long-term studies concerning this, with no general consensus.
